# Diversity of *Mycobacteriaceae* from aquatic environment at the São Paulo Zoological Park Foundation in Brazil

**DOI:** 10.1371/journal.pone.0227759

**Published:** 2020-01-14

**Authors:** Camila Lopes Romagnoli, Katia Cristina Machado Pellegrino, Natalia Maria Silva, Urze Adomaitis Brianesi, Sylvia Cardoso Leão, Michelle Christiane da Silva Rabello, Cristina Viana-Niero

**Affiliations:** 1 Departamento de Microbiologia, Imunologia e Parasitologia da Universidade Federal de São Paulo, São Paulo, SP, Brazil; 2 Departamento de Ecologia e Biologia Evolutiva da Universidade Federal de São Paulo, Diadema, SP, Brazil; 3 Departamento de Imunologia do Instituto Aggeu Magalhães, Fundação Oswaldo Cruz, Recife, PE, Brazil; UFRJ, BRAZIL

## Abstract

We investigated the species diversity of *Mycobacteriaceae* in surface water samples from six environments at the zoological park in São Paulo, Brazil. Three hundred and eighty isolates were cultivated and identified by phenotypic characteristics (growth rate and pigmentation) and sequencing of *hsp*65, *rpo*B and 16S rRNA genes. The results revealed that almost 48% of the isolates could be identified at the species level; about 50% were classified at the genus level, and only less than 2% of the isolates showed an inconclusive identification. The isolates classified at the genus level and not identified were then evaluated by phylogenetic analyses using the same three concatenated target genes. The results allowed us to identify at the genus level some isolates that previously had inconclusive identification, and they also suggested the presence of putative candidate species within the sample, demonstrating that this zoological park is an important source of diversity.

## Introduction

A comparative genomic analysis among 150 species of the genus *Mycobacterium* based on core proteins has shown the existence of distinct monophyletic groups, leading to the division of the genus into five distinctive genera [1,2]. According to this study, the genera of the family *Mycobacteriaceae* were named *Mycobacterium*, which includes all of the major human pathogens, namely *Mycolicibacterium*, *Mycolicibacter*, *Mycolicibacillus* and *Mycobacteroides*, corresponding to the “Fortuitum-Vaccae”, “Terrae”, “Triviale” and “Abscessus-Chelonae” clades, respectively.

Most species of the family *Mycobacteriaceae* are considered saprophytic microorganisms and inhabitants of several natural environments, such as lakes, rivers, swamps, soils and environments influenced by humans, such as water treatment and distribution systems [3–7]. Some environmental isolates have raised interest because of their ability to metabolize aliphatic hydrocarbons and polycyclic aromatic hydrocarbons, which are important pollutants [8–11]. Apart from that, genome analysis of *M*. *brisbanense* (UM_WWY) has revealed the presence of genes associated with nitrogen and urea cycles, suggesting that their use in soil may result in the synthesis of urea, favoring plant growth [12].

In Brazil, studies on the diversity of environmental isolates from *Mycobacteriaceae* are still scarce. Lima-Junior *et al*. (2016) characterized isolates of *Mycobacteriaceae* and mycobacteriophages from the compost at São Paulo Zoo and isolated an environmental *M*. *insubricum* for the first time [13]. The mycobacteriophage isolates displayed considerable genomic diversity and the phage Madruga showed over 95% similarity with the genome of the phage Patience forming a new assigned cluster U. These data demonstrate the richness of the microbial communities within the composting systems and their potential source for the prospecting of new phage and bacterial isolates. The São Paulo Zoological Park Foundation (FPZSP) has an area of 824,529 m^2^ of original Atlantic Forest, and besides the composting unit, it has several springs and lakes that shelter captive and free-living animals and migratory birds, a water treatment station and a sewage treatment station.

This work aimed to isolate microorganisms from the family *Mycobacteriaceae* from six aquatic environments with distinct anthropic and trophic characteristics at the zoo in São Paulo, Brazil. We investigated the diversity of *Mycobacteriaceae* isolates and found the possible occurrence of putative candidate species among these isolates.

## Material and methods

### Description of study sites

FPZSP is located in an urban area of the city of São Paulo, and within a remnant area of the Atlantic Forest. Its location is between the parallels 23° 38’ 08” S and 23° 40’ 18” S and the meridians 46°36’48” W and 46°38’00” W. Surface water samples from six environments of FPZSP were studied, which were classified according to the degree of anthropization, color and odor as suggested by Bicudo *et al*., 2002 [14]. The Bigger Spring (BS) has an area of 3,852 m^2^, and is odorless and barren; it is an enclosure for five alligators and has little human influence. The Smaller Spring (SS) has a water area of 388 m^2^ and is clear and odorless; it is located inside a bird enclosure, where it is considered an environment with a medium degree of anthropization. Lake70 (L70) has an area of 34,665 m^2^ and is an artificial lake with a medium degree of anthropization; it is inhabited by primates and local and migratory birds. A particular feature of this environment is the presence of algae, making the water greenish though odorless. Water samples from the sewage treatment station were also analyzed at two points, the crude sewage (cS) and treated sewage (tS). The sewage treatment station receives water from the animal enclosures and the daily activities of the park, which has a characteristic odor and brownish color. Water from the water treatment station (WTS) was collected shortly after the end of the physical and chemical treatment. WTS treats water from the sewage treatment station and Lake70 on alternate days, generating re-cycled water to wash the animal enclosures and also to restore the water level of Lake70. This study was approved by the Research Ethics Committee of UNIFESP and FPZSP under numbers 0082/10 and 263/2009, respectively.

### Sample collection

Surface water samples were collected from six different sources every month between November 2011 and October 2012, totaling 72 samples. Using a sterile flask, 1L of water was collected from each site, and for the WTS water the flasks contained 0.01% sodium thiosulfate for neutralization of residual chlorine [15]. The samples were stored and transported at 4°C until processed, which did not exceed three hours.

### Processing of water samples

Each sample was totally concentrated under vacuum and filtered with a 0.45-μm nitrocellulose membrane (Millipore) for subsequent decontamination and culture of bacteria belonging to the family *Mycobacteriaceae*. The water samples from Lake70 were first filtered with a 20-μm mesh phytoplankton net and then with a 8-μm glass microfiber filter (AP20 Millipore), followed by filtration through a 5-μm cellulose ester membrane (Millipore) for the removal of microalgae [16–18]. The crude and treated sewage samples were also previously pre-filtered due to a high concentration of organic matter.

After the sample concentration procedure, each membrane was transferred to a sterile tube with 10 mL of phosphate-buffered saline (PBS 1X, pH 7.4) and maintained under vigorous stirring for 30 min. The bacterial suspension was decontaminated with 0.05% cetylpyridinium chloride (CPC) and spread on Middlebrook 7H10 medium supplemented with 0.5% glycerol and 10% OADC-oleic acid, albumin, dextrose and catalase (Becton Dickinson) and 10% PANTA (40 U/mL polymyxin, 4 μg/mL amphotericin B, 16 μg/mL nalidixic acid, 4 μg/mL trimethoprim and 4 μg/mL azlocillin) (Becton Dickinson) according to the protocol described by Radomski *et al*., 2010 [19]. The cultures were incubated at 30°C and examined for 60 days.

### Species identification

All recovered colonies were initially visualized under a light microscope (Eclipse E100, Nikon) after Ziehl-Neelsen staining (Renylab, Brazil). Acid-fast bacteria colonies were isolated and identified by evaluation of phenotypic characteristics (growth rate and pigment) and sequences of the *hsp65* gene, *rpoB* gene V region and 16S rRNA gene [20–24].

DNA was extracted by thermal lysis, that is suspension of bacterial colonies in 300 μL of sterile MiliQ water and incubation at 95°C for 10 minutes, and 5 μL of the supernatant were used for each PCR. The regions amplified by PCR included a 667 bp fragment of *hsp65* gene, containing the fragment described by Telenti, a 752 bp fragment of the V region of *rpoB* and the complete (1,500 bp) 16S rRNA gene [20,22–25]. The amplification and sequencing of region III of *rpoB* gene performed in cases of doubt in the identification [26]. The amplicons were purified using the QIAquick PCR purification kit (Qiagen), as recommended by the manufacturer and were sequenced in both directions in an ABI Prism 3500xL Sequencer (Applied Biosystems, Foster City, CA, USA). The primers used for sequencing reactions were the same as those for PCR, except for the sequencing of the 16S rRNA gene, which included the internal primers described by Adékambi & Drancourt (2004) [23].

Nucleotide sequences were edited using the BioNumerics program version 7.6.3 (Applied Maths, Sint-Martens-Latem, Belgium) to generate single consensus sequences, which were later compared with those deposited in the NCBI database (National Center for Biotechnology Information) using the tool BLAST–Basic Local Alignment (URL: http://www.ncbi.nlm.nih.gov/BLAST). The cut-off points for the analyses of the *hsp65*, *rpoB* and 16S rRNA genes were ≥97, ≥98.3 and ≥99%, respectively [22,27,28].

Consensus sequences of *rpoB* and 16S rRNA gene regions were aligned using Bionumerics 7.6.3 with Fast Algorithm (AppliedMaths, Sint-Martens-Latem, Belgium) and those of *hsp65* using Clustal W 1.6 [29], implemented on Mega 7.0 [30]. Alignments of coding regions (*hsp65* and *rpoB*) were translated into amino acids using the program Se-Al v.2.0a11 (http://tree.bio.ed.ac.uk/software/seal) to verify the presence of unexpected stop-codons. One representative of each sequence obtained from the *hsp65*, *rpoB* and 16S rRNA genes were deposited in GenBank under accession numbers KP768386, KP768387, KR779718, MK876855-MK877231; MK879838-MK879916; KP768388, KP768389, KR779819, and MK890454-890532.

### Phylogenetic analysis

The isolates identified at the genus level according to Gupta *et al*, (2018) [1] along with unidentified isolates and 86 sequences of described *Mycobacteriaceae* species downloaded from GenBank (S1 Table) were used in the phylogenetic analyses. The species *Corynebacterium afermentans* DSM 44280 (NZ_FTMH00000000.1), *Nocardia abscessus* DSM44557 (JN041731.1; JN215598.1; JN041494.1) and *Tsukamurella paurometabola* NCTC13232 (NZ_UHIQ00000000.1) were selected as outgroups.

Phylogenetic analyses were performed using a concatenated dataset of 2,511 bp from the two fragments of the coding gene regions of *hsp65* and *rpoB* and the ribosomal 16S rRNA gene. Prior to analyses, models of substitution for each gene region and the concatenated dataset were selected using jModelTest v.2.1.6 [31], according to Akaike Information Criterion (AIC) as implemented at CIPRES Science Gateway (https://www.phylo.org/).

Phylogenetic analyses were carried out using both maximum-likelihood (ML) and bayesian inference (BI). The tree search under the ML criterion was performed in PAUP v.4.0 with 20 replicates of random stepwise addition under tree-bisection-reconnection (TBR) [32]. BI analyses were conducted in MRBAYES v.3.2.2 [33] implemented at CIPRES Science Gateway (https://www.phylo.org/), with two independent searches of 10^7^ generations each, sampling every 1000 generations, and with four Markov Chain Monte Carlo (MCMC) runs. We assessed convergence between runs using Tracer v1.6 [34] and discarded the first 20% trees as burn-in.

Node support in resulting nodes on ML tree was evaluated by 200 non-parametric bootstrap analyses and by posterior probability values the BI topology [35]. Nodes with bootstrap proportions (BP) ≥ 70% ([36]; with caveats) on the ML tree and those with posterior values (PP) ≥ 0.95 on BI tree were considered as evidence of supported clades [33]. Trees were edited in the program FigTree 1.3.1. (http://tree.bio.ed.ac.uk/).

### Analysis of the diversity of species

The species diversity in each environment studied was analyzed by the Shannon-Wiener index (H’) [37]. This index determines the alpha (α) diversity of a local community providing values that allow the comparison of diversity between the different sites sampled.

## Results

### Identification of isolates

**Fig 1:**
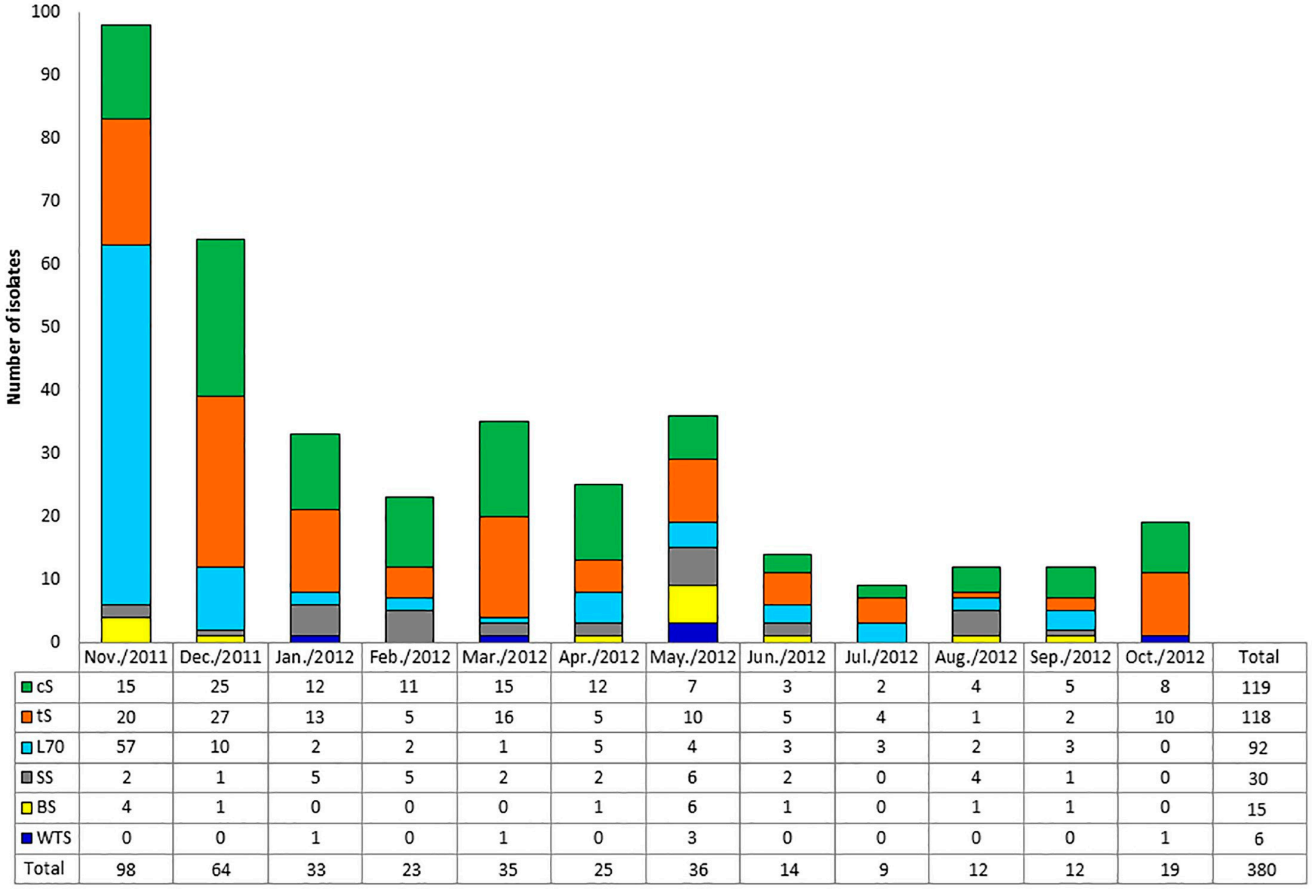
Distribution of *Mycobacteriaceae* isolates according to origin and period of analysis. cS: crude sewage; tS: treated sewage; L70: Lake70; SS: Smaller Spring; BS: Bigger Spring and WTS: water treatment station.

Among the 72 water samples analyzed, 56 (77.7%) showed growth of *Mycobacteriaceae* totaling 380 isolates. A total of 237 isolates were collected from the effluents, 92 from Lake70, 45 from the springs and 6 from the water treatment station, Fig 1.

Most of the isolates (314, 82.6%), were recovered during the months of November to May, corresponding to the period of higher temperatures in Brazil, Fig 1. According to the phenotypic classification, 347 isolates (91.3%) displayed rapid growth and 33 (8.7%) slow growth. Regarding pigmentation, 214 (56.3%) isolates were classified as achromogenic and 166 (43.7%) as chromogenic.

**Fig 2:**
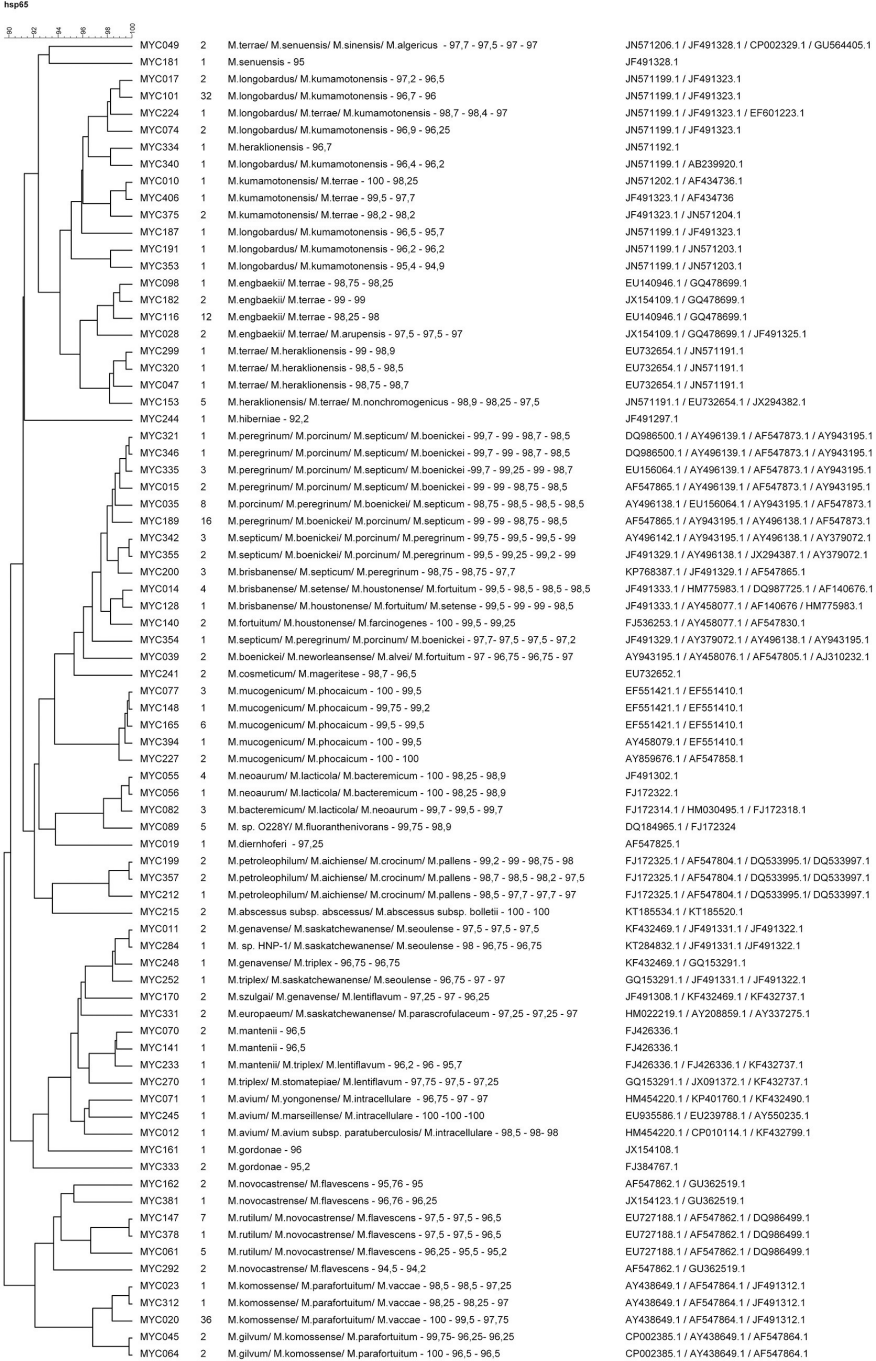
Classification of the isolates into 78 groups by sequencing the *hsp65* gene. Similarity dendrogram was based on the multiple alignment matrix of the *hsp65* gene sequences using the UPGMA method. In the first column are the isolates randomly selected to be analyzed by sequencing the 16S rRNA and *rpoB* genes. Column 2: Number of isolates included in each group. Column 3: Result of the comparison of the *hsp65* gene sequences of isolates with sequences deposited in the GenBank database with their percentages of identity. Column 4: GenBank accession number of each sequence.

Initially, the identification was performed by sequencing a 401 bp fragment of *hps65* gene for all isolates, and the results were analyzed together with phenotypic characteristics. All sequences were deposited in GenBank as described in the Material and Methods section. Thus, it was possible to identify 140 isolates (36.8%) corresponding to the species *Mycobacteroides chelonae*, *Mycobacterium gordonae*, *Mycobacterium parascrofulaceum*, *Mycobacterium lentiflavum*, *Mycobacterium kansasii*, *Mycolicibacter arupensis*, *Mycolicibacterium insubricum* and *Mycolicibacterium austroafricanum*/*M*. *vanbaalenii*. In addition, these results allowed us to classify the other 240 isolates (63.2%) as belonging to the genera *Mycobacterium*, *Mycobacteroides*, *Mycolicibacterium* and *Mycolicibacter* and also to separate them into 78 groups, Fig 2.

To further identify these isolates, at least one representative of each group was randomly selected and then sequenced for the 16S rRNA and *rpo*B genes.

The analysis of the three targets allowed us to identify 11.1% more isolates to the species level, namely: *Mycobacteroides abscessus* subsp. *bolletii*, *Mycolicibacterium bacteremicum*, *Mycolicibacterium brisbanense*¸ *Mycolicibacterium fluoranthenivorans*, *Mycolicibacterium fortuitum*, *Mycolicibacterium mucogenicum*, *Mycolicibacterium neoaurum*, *Mycolicibacterium phocaicum*, *Mycolicibacterium septicum*, *Mycolicibacter kumamotonensis* and *Mycobacterium marseillens*e. Thus, at the end of the identification stage, 182 (47.9%) isolates were identified at the species level, 191 (50.3%), clustered in 55 groups, were classified at the genus level, while seven isolates (1.8%), clustered in 6 groups, showed identity with sequences of species belonging to different genera, so their identification was considered inconclusive, Table 1.

**Table 1:** Identification of isolates and distribution according to site of origin. cS: crude sewage; tS: treated sewage; L70: Lake70; SS: Smaller Spring; BS: Bigger Spring and WTS: water treatment station.

	cS	tS	L70	SS	BS	WTS	Total (%)
**Species identified**							
*Mycobacterium gordonae*	9	11	10	12	-	-	42 (11,1%)
*Mycolicibacterium insubricum*	1	31	1	-	1	-	34 (8,9%)
*Mycobacteroides chelonae*	4	-	8	8	7	3	30 (7,9%)
*Mycobacterium parascrofulaceum*	3	11	-	-	-	-	14 (3,7%)
*Mycolicibacterium mucogenicum*	8	3	-	-	-	-	11 (2,9%)
*Mycobacterium kansasii*	-	-	8	-	-	-	8 (2,1%)
*Mycolicibacter arupensis*	6	-	1	-	-	-	7 (1,8%)
*Mycolicibacterium austroafricanum/vanbaalenii*	-	4	1	-	-	-	5 (1,3%)
*Mycolicibacterium brisbanense*	5	-	-	-	-	-	5 (1,3%)
*Mycolicibacterium fluoranthenivorans*	1	1	3	-	-	-	5 (1,3%)
*Mycolicibacterium neoaurum*	-	-	5	-	-	-	5 (1,3%)
*Mycolicibacterium bacteremicum*	-	3	-	-	-	-	3 (0,8%)
*Mycolicibacterium septicum*	1	-	-	1	-	1	3 (0,8%)
*Mycobacteroides abscessus subsp*. *bolletti*	2	-	-	-	-	-	2 (0,5%)
*Mycolicibacterium fortuitum*	2	-	-	-	-	-	2 (0,5%)
*Mycobacterium lentiflavum*	1	-	-	-	-	1	2 (0,5%)
*Mycolicibacterium phocaicum*	-	-	1	1	-	-	2 (0,5%)
*Mycolicibacter kumamotonensis*	1	-	-	-	-	-	1 (0,3%)
*Mycobacterium marseillense*	-	1	-	-	-	-	1 (0,3%)
**Genus identified**							
*Mycolicibacterium*	32	20	49	2	3	1	107 (28,2%)
*Mycolicibacter*	40	27	2	3	1	-	73 (19,2%)
*Mycobacterium*	2	1	4	2	2	-	11 (2,9%)
**Not identified**							
	2	4	-	1	-	-	7 (1,8%)
Total	119	118	92	30	15	6	380 (100%)

The species *Mycobacterium gordonae*, *Mycolicibacterium insubricum* and *Mycobacteroides chelonae* were the most prevalent, representing between 11 and 7.9% of the isolates identified. The species *Mycobacterium parascrofulaceum*, *Mycolicibacterium mucogenicum*, *Mycobacterium kansasii*, *Mycolicibacter arupensis*, *Mycolicibacterium austroafricanum*/*vanbaalenii*, *Mycolicibacterium fluoranthenivorans*, *Mycolicibacterium neoaurum*, *Mycolicibacterium brisbanense*, *Mycolicibacterium bacteremicum*, *Mycolicibacterium septicum*, *Mycobacteroides abscessus subsp*. *bolletti*, *Mycobacterium lentiflavum*, *Mycobacterium marseillense* and *Mycolicibacter kumamotonsensis* were also found in the samples analyzed and represented between 3.7 and 0.3% of the total isolates. *Mycolicibacterium* was the most prevalent genus, followed by *Mycolicibacter* and *Mycobacterium*.

A more detailed analysis of the isolates from Lake70 revealed that 36 of the 49 isolates were recovered in November and December 2011. They were initially identified as *Mycolicibacterium komossense*, because they showed pigment, rapid growth and sequence identity of 100 and 98.7% with *hsp65* and 16S rRNA genes of that species (AY438649.1; NR026086.1), respectively. The sequence of *rpoB*V gene of *M*. *komossense* was not available in databases, and thus, *rpoB* region III sequencing was performed here (Genbank accession number MK907678), which showed 93.9 and 98.2% identity with the sequences of *M*. *komossense* (AY544936.1) and *Mycolicibacterium parafortuitum* (AY544952.1), raising doubts about their identification.

### Phylogenetic analysis

The phylogenetic analyses were conducted on a concatenated dataset of sequences from three genes (*hsp65*, *rpoB* and ribosomal 16S rRNA), comprising 2,511 bp of 152 members of *Mycobacteriaceae* that included 60 isolates (representing 55 groups classified at the genus level), six isolates with inconclusive identification and 86 sequences from known species (S1 Table) analyzed with both ML and BI methods. Groups with more than 15 isolates had more than one isolate analyzed.

**Fig 3:**
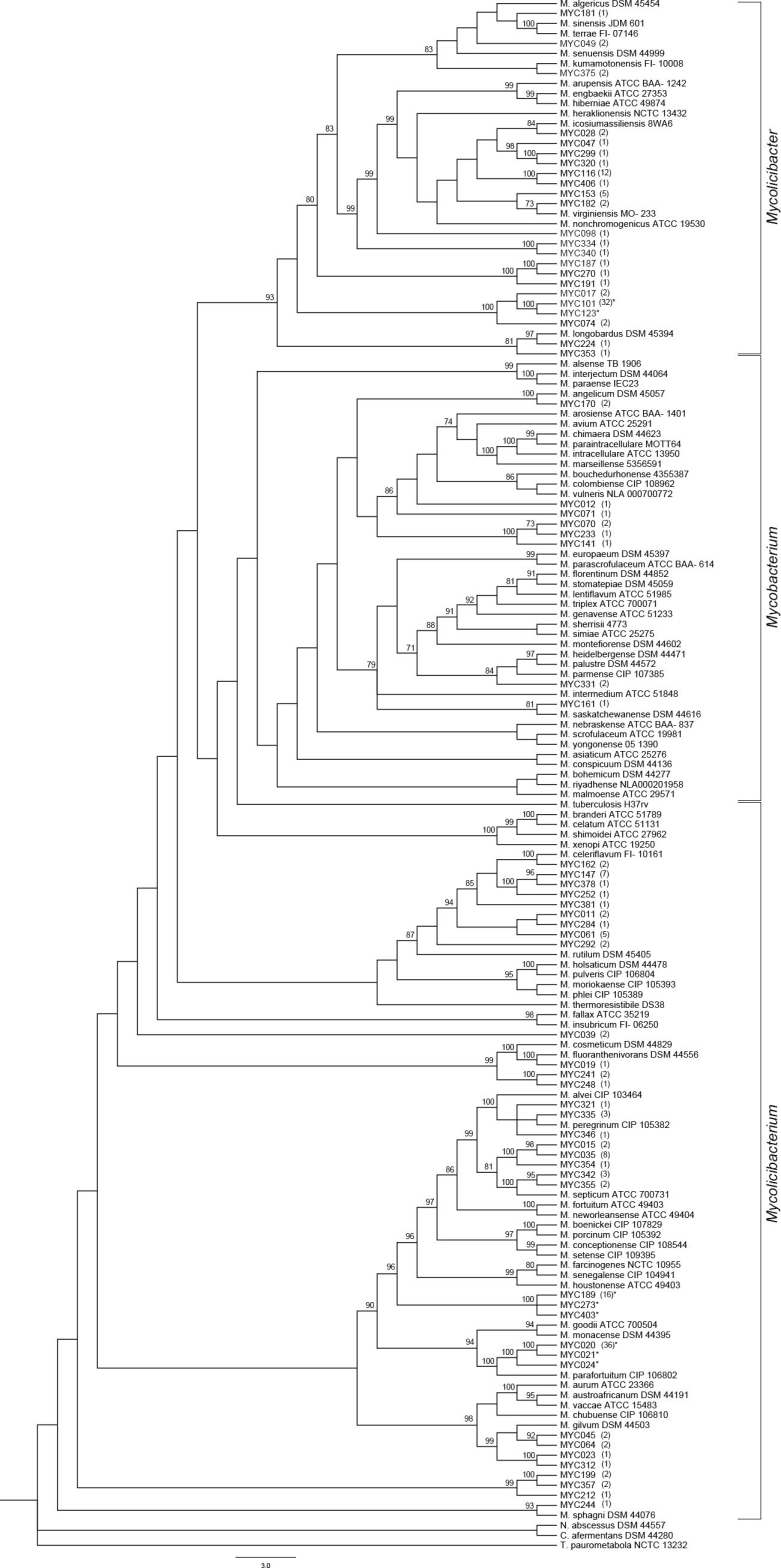
Phylogeny of *Mycobacteriaceae* based on concatenated sequences of *hsp65*, *rpoB* and 16S rRNA genes (2,511 bp), according to maximum-likelihood method (Lnl = 36046.26). Values above branches are bootstrap proportions (≥70%) and those in parenthesis after the terminal names refer to number of isolates belonging to each group. * Groups with more than one isolate studied by phylogenetic analysis.

ML analysis was conducted under the GTR (general time-reversible) model of evolution, with rate of variable sites (gamma = 0.547) and the proportion of invariant sites (I = 0.545), and generated 3 equally likely trees, from which we produced a semi-strict consensus tree (Lnl = 36046.26; Fig 3). BI analysis (*hsp65*: GTR+I+G; *rpoB*: TIM1+G; 16S rRNA: TIM3+I+G) produced a very similar topology as the ML consensus tree (S1 Fig).

These phylogenetic analyses allowed us to identify at the genus level some isolates whose identification was previously considered inconclusive, such as MYC270 belonging to *Mycolicibacter* and isolates MYC252, MY011, MYC284, MYC248 and MYC244 attributed to the genus *Mycolicibacterium* (Fig 3 and S1 Fig).

Herein, we focused our attention on groups of isolates of the genera *Mycolicibacter* and *Mycobacterium*, which may contain potentially candidate species. Within the genus *Mycolicibacter*, some isolates were grouped closely related to sequences of known species: MYC028 to *Mycolicibacter icosiumassiliensis* (BP = 84, PP = 0.88), MYC182 to *Mycolicibacter virginiensis* (BP = 73, PP = 0.76), and MYC224 to *Mycolicibacter longobardus*, (BP = 97, PP = 1.0), with strong support, especially with the ML method (bootstrap proportions). Regarding isolates MYC049 and MYC375, both ML and BI methods uncovered different relationships between them and other known species, some without support.

Contrarily, ten isolates placed in the genus *Mycolicibacter* were recovered in distinct phylogenetic groups and more distantly related to those sequences of known species. For instance, the isolate MYC098 was recovered externally to a major group that contains seven known species and eight isolates (BP = 99; PP = 0.99; Fig 3 and S1 Fig). Next in the topology, the highly supported sister group MYC334+MYC340 (BP = 100; PP = 0.99) was placed. Externally, the clade containing (MYC187+ MYC270) with MYC191 as its sister taxon (BP = 100; PP = 1.0) was recovered. Following all these clades, a major clade was recovered (BP = 100; PP = 1.0) grouping MYC101 and MYC123 (BP = 100; PP = 1.0) with MYC017 (BP<50; PP = 0.73) as its sister group, and isolate MYC074 placed basal to them (BP = 100; PP = 0.99).

Within the genus *Mycobacterium*, MYC170 was recovered as sister to *Mycobacterium angelicum* (BP = 100; PP = 0.99), while MYC161 grouped with *Mycobacterium saskatchewanense* (BP = 81; PP = 0.84; Fig 3 and S1 Fig). As a distinct group, both ML and BI methods recovered a clade formed by (MYC070+MYC233; BP = 73, PP = 0.74) with MYC141 sister to them (BP = 100; PP = 1.0).

Additionally, we mention that within the genus *Mycolicibacterium* our phylogenetic analyses recovered a well-supported major clade containing five isolates (MYC015+MYC035+MYC354) and (MYC342+MYC355), the last being placed as sister to the *M*. *septicum* species (BP = 81; PP = 0.796; Fig 3 and S1 Fig).

### Analysis of bacterial diversity

After the identification of the isolates, it was possible to evaluate the bacterial diversity by origin. The values of α diversity found were: 3.28 for crude sewage (cS), 2.73 for treated sewage (tS), 2.31 for Lake70 (L70) and 1.7, 1.65 and 1.25 for Bigger Spring (BS), Smaller Spring (SS) and the water treatment station (WTS), respectively. These results revealed that the cS, tS and L70 environments had higher diversity than the others investigated.

## Discussion

In the last decades, we have observed an increase in *Mycobacteriaceae* species described. This study aimed to determine the diversity of these bacteria in water samples in a zoological park during a period of twelve months.

Most of the isolates were recovered during the warmest period of the year and also from eutrophic environments, in accordance with data already described in other studies [38–41].

Using an approach associated with sequencing three genes and phenotypic characteristics, we were able to characterize almost 48% of the isolates at the species level and 50.3% at the genus level. Several studies have described that analyses based on concatenated housekeeping genes support the clustering of most species in well-defined phylogenetic groups and emphasize the importance of including also data on growth rate and pigmentation of colonies [1,23,42–46].

*M*. *fluoranthenivorans* DSM44556, *M*. *vanbaalenii*-PYR1 and *M*. *neoaurum* NRRL B-3805 were isolated from the environment by screening tests and later characterized as sterol, hydrocarbon and aflatoxin B degraders, indicating potential biotechnological application [47–49]. Among the species identified herein, there are saprophytic and potentially pathogenic ones and also species previously described as having biotechnological potential. In addition, it was possible to isolate *M*. *bacteremicum*, a species involved in cases of catheter-related sepsis, in samples from the sewage treatment plant, where this is the first report of isolation of this species from water samples [50]. A curious finding is that the isolates identified as *M*. *komossense* were prevalent in Lake70 only during the months of November and December. These isolates exhibited more similarity to *M*. *parafortuitum* than to *M*. *komossense* on the basis of the region III of *rpoB* gene sequence. Kim and colleagues (2013) reported the horizontal transfer of the *rpoB* gene from *M*. *parascrofulaceum* to *Mycobacterium yongonense* [51]. A horizontal gene transfer event may have occurred in the isolates of this study, and it is a hypothesis that would explain our results.

Our study showed that all analyzed aquatic environments at the zoo presented several species of *Mycobacteriaceae* and, many of them might have been characterized at genus level without conclusive species identification, possibly belonging to new taxa. Some studies have demonstrated that inconclusive results after the analysis of several genes are indicative of putative new species and also point to the importance of a phylogenetic approach to achieve this conclusion [50,52–54].

The results of our phylogenetic analyses based on three concatenated target genes confirmed the identity of isolates at the genus level. Also, this allowed us to verify the relationships within the *Mycolicibacterium* and *Mycobacterium* clades, as isolates MYC028, MYC182 and MYC224 are closely related to *M*. *icosiumassiliensis*, *M*. *virginiensis* and *M*. *longobardus*, and MYC170 and MYC161 recovered closely to *M*. *angelicum* and *M*. *saskatchewanense*, respectively. Besides, among the isolates with inconclusive identification, the ML and BI phylogenies made it possible to recognize one isolate as belonging to the genus *Mycolicibacter* and to attribute six others to the genus *Mycobacterium*, confirming the value of inferring phylogenetic relationships for more accurate identification of *Mycobateriaceae*. The present work suggests the presence of putative candidate species.

Some studies on microbial diversity use metagenome analyses since they consider that cultivation approaches provide a limited view on bacterial diversity [41,55]. However, the analysis of gut microbiota based on different cultivation conditions (microbial culturomics) has allowed the isolation and description of unknown species within the human intestine, reinforcing the importance of studies supported by bacterial culture [56–58]. In this work, we demonstrated the possibility of using decontamination and cultivation methods to recover environmental isolates representing species diversity in the family *Mycobacteriaceae*. Isolation of bacteria presents the advantage of having the live isolates, and not only their sequences, for genetic, physiological, taxonomic studies and biotechnological applications.

## Supporting information

S1 Table List of *hsp65*, *rpoB* and 16S rRNA sequences of *Mycobacteriaceae* species downloaded from GenBank used in the phylogenetic analyses. (DOCX)

S1 Fig Bayesian phylogeny based on concatenated sequences of *hsp65*, *rpoB* and 16S rRNA genes (2,511 bp). Numbers above branches are Bayesian posterior probabilities (≥0.95).
(TIF)
