# Supplementary material for: Diversity of Mycobacteriaceae from aquatic environment at the São Paulo Zoological Park Foundation in Brazil
Source: PLoS One. 2020 Jan 14;15(1):e0227759. doi: 10.1371/journal.pone.0227759 (PMC6959594; doi:10.1371/journal.pone.0227759)
Supplement: S1 Table — (DOCX) [file pone.0227759.s001.docx]

| Species | *hsp65* | 16S rRNA | *rpoB* |
| --- | --- | --- | --- |
| *Mycolicibacter algericus* DSM 45454 | GU564405.1 | GU564404.1 | GU564406.1 |
| *Mycobacterium alsense* TB 0906 | FM209298.1 | NR147779.2 | KT168292.1 |
| *Mycolicibacterium alvei* CIP 103464 | AF547805.1 | AF547895.1 | AY859697.1 |
| *Mycobacterium angelicum* DSM 45057 | AM902962.2 | NR145908.1 | KT168289.1 |
| *Mycobacterium arosiense* ATCC BAA-1401 | GQ153297.1 | NR117221.1 | GQ153312.1 |
| *Mycolicibacter arupensis* ATCC BAA-1242 | JN571186.1 | JN571167.1 | KT861786.1 |
| *Mycobacterium asiaticum* ATCC 25276 | AY299133.1 | NR041901.1 | KT168291.1 |
| *Mycolicibacterium aurum* ATCC 23366 | AY299154.1 | FJ172298.1 | FJ172341.1 |
| *Mycolicibacterium austroafricanum* DSM 44191 | AF547807.1 | NR026284.1 | HG964450.1 |
| *Mycobacterium avium* ATCC 25291 | GQ153289.1 | NR025584.1 | EF521907.1 |
| *Mycolicibacterium boenickei* CIP 107829 | AY943195.1 | NZFUWC01000003.1 | NZFUWC01000003.1 |
| *Mycobacterium bohemicum* DSM 44277 | AF547811.1 | NR026054.1 | KJ729110.1 |
| *Mycobacterium bouchedurhonense* 4355387 | EU239790.1 | NR116063.1 | EF584445.1 |
| *Mycobacterium branderi* ATCC 51789 | AF547815.1 | NR041897.1 | JQ599259.2 |
| *Mycobacterium celatum* ATCC 51131 | AY299181.1 | NR029143.1 | JF346871.1 |
| *Mycolicibacterium celeriflavum* FI-10161 | KJ586614.1 | KJ586590.1 | KJ586585.1 |
| *Mycobacterium chimaera* DSM 44623 | EU239783.1 | EF521894.1 | EF521908.1 |
| *Mycolicibacterium chubuense* CIP 106810 | AF547821.1 | NR041902.1 | UATB01000015.1 |
| *Mycobacterium colombiense* CIP 108962 | GQ153298.1 | NR117222.1 | EF521910.1 |
| *Mycolicibacterium conceptionense* CIP 108544 | AY859678.2 | AY859684.1 | AY859695.1 |
| *Mycobacterium conspicuum* DSM 44136 | AF547823.1 | AF547914.1 | HQ141573.1 |
| *Mycolicibacterium cosmeticum* DSM 44829 | DQ124111.1 | MH169226.1 | CCBB010000003.1 |
| *Mycolicibacter engbaekii* ATCC 27353 | JN571196.1 | NR114655.1 | JN571244.1 |
| *Mycobacterium europaeum* DSM 45397 | HM022220.1 | NR125568.1 | HM022215.1 |
| *Mycolicibacterium fallax* ATCC 35219 | JF491294.1 | AF480600.1 | EU999023.1 |
| *Mycolicibacterium farcinogenes* NCTC 10955 | AY458073.1 | LT718447.1 | AY262742.1 |
| *Mycobacterium florentinum* DSM 44852 | JF491317.1 | NR042223.1 | HM022205.1 |
| *Mycolicibacterium fluoranthenivorans* DSM 44556 | JF491318.1 | FJ172310.1 | FJ172339.1 |
| *Mycolicibacterium fortuitum* ATCC 49403 | AY299153.1 | AF480580.1 | AY147173.1 |
| *Mycobacterium genavense* ATCC 51233 | AY299183.1 | NR029223.1 | HM022216.1 |
| *Mycolicibacterium gilvum* DSM 44503 | UGQM01000001.1 | NR118915.1 | UGQM01000001.1 |
| *Mycolicibacterium goodii* ATCC 700504 | AY458071.1 | AY457079.1 | AY262736.1 |
| *Mycobacterium heidelbergense* DSM 44471 | AF547844.1 | AF547935.1 | HM132044.1 |
| *Mycolicibacter heraklionensis* NCTC 13432 | JN571192.1 | NR117431.1 | JN571231.1 |
| *Mycolicibacter hiberniae* ATCC 49874 | JN571198.1 | NZLQOZ01000024.1 | JN571246.1 |
| *Mycolicibacterium holsaticum* DSM 44478 | AY438084.1 | NR028945.2 | AY859705.1 |
| *Mycolicibacterium houstonense* ATCC 49403 | AY458077.1 | LT223693.1 | AY147173 |
| *Mycolicibacter icosiumassiliensis* 8WA6 | KT592293.1 | KT592291.1 | KT592292.1 |
| *Mycolicibacterium insubricum* FI-06250 | EF584487.1 | EU605695.1 | EU022519.1 |
| *Mycobacterium interjectum* DSM 44064 | AF547846.1 | LT223694.1 | HM022207.1 |
| *Mycobacterium intermedium* ATCC 51848 | AY299187.1 | AF547938.1 | JF712874.1 |
| *Mycobacterium intracellulare* ATCC 13950 | GQ153290.1 | NR117223.1 | GQ153307.1 |
| *Mycolicibacter kumamotonsensis* FI-10008 | JN571203.1 | JN571179.1 | JN571258.1 |
| *Mycobacterium lentiflavum* ATCC 51985 | AY373453.1 | NR041898.1 | JN881350.1 |
| *Mycolicibacter longobardus* DSM 45394 | JN571199.1 | JN571166.1 | JN571247.1 |
| *Mycobacterium malmoense* ATCC 29571 | GQ153293.1 | NR117225.1 | GQ153314.1 |
| *Mycobacterium marseillense* 5356591 | EU239787.1 | NR116262.1 | EF584434.1 |
| *Mycolicibacterium monacense* DSM 44395 | JF491320.1 | NR041723.1 | KU361327.1 |
| *Mycobacterium montefiorense* DSM 44602 | AY943204.1 | NR028808.1 | HM022209.1 |
| *Mycolicibacterium moriokaense* CIP 105393 | AF547857.1 | AY859686.1 | AY859699.1 |
| *Mycobacterium nebraskense* ATCC BAA-837 | GQ153294.1 | NR117224.1 | GQ153310.1 |
| *Mycolicibacterium neworleansense* ATCC 49404 | AY458076.1 | AY457068.1 | CWKH01000001.1 |
| *Mycolicibacter nonchromogenicus* ATCC 19350 | AY299136.1 | NR044813.1 | JN881351.1 |
| *Mycobacterium palustre* DSM 44572 | AY943200.1 | AY943210.1 | HM022210.1 |
| *Myobacterium paraense* IEC23 | KJ949035.1 | KJ948993.1 | KJ949007.1 |
| *Mycolicibacterium parafortuitum* CIP 106802 | AF547864.1 | NR026285.1 | UEGS01000001.1 |
| *Mycobacterium paraintracellulare* MOTT64 | KP670342.1 | KP670329.1 | KP890661.1 |
| *Mycobacterium parascrofulaceum* ATCC BAA-614 | AY337274.1 | NR117220.1 | GU139240.1 |
| *Mycobacterium parmense* CIP 107385 | HM022199.1 | HM022201.1 | HM022211.1 |
| *Mycolicibacterium peregrinum* CIP 105382 | AY458069.1 | AY457069.1 | AY147166.1 |
| *Mycolicibacterium phlei* CIP 105389 | AF547866.1 | NR041906.1 | AY859700.1 |
| *Mycolicibacterium porcinum* CIP 105392 | AY458068.1 | AY457077.1 | AY262737.1 |
| *Mycolicibacterium pulveris* CIP 106804 | AF547869.1 | NR025528.1 | AY859701.1 |
| *Mycobacterium riyadhense* NLA000201958 | EU921671.1 | NR044449.1 | FJ786256.1 |
| *Mycolicibacterium rutilum* DSM 45405 | LT629971.1 | LT629971.1 | LT629971.1 |
| *Mycobacterium saskatchewanense* DSM 44616 | JF491331.1 | NR042793.1 | HM022212.1 |
| *Mycobacterium scrofulaceum* ATCC 19981 | GQ153288.1 | NR025237.1 | GQ153305.1 |
| *Mycolicibacterium senegalense* CIP 104941 | AY458067.1 | AY457081.1 | AY262738.1 |
| *Mycolicibacter senuensis* DSM 44999 | JN571200.1 | FJ268583.1 | JN571249.1 |
| *Mycolicibacterium septicum* ATCC 700731 | AY373457.1 | AY457070.1 | AY147167.1 |
| *Mycolicibacterium setense* CIP 109395 | EU371505.1 | EU371504.1 | EU371506.1 |
| *Mycobacterium sherrisii* 4773 | AY365190.1 | NR115209.1 | KJ920345.1 |
| *Mycobacterium shimoidei* ATCC 27962 | AY299140.1 | NR041945.1 | HM807416.1 |
| *Mycobacterium simiae* ATCC 25275 | GQ153292.1 | NR117227.1 | GQ153313.1 |
| *Mycolicibacter sinensis* JDM 601 | CP002329.1 | CP002329.1 | CP002329.1 |
| *Mycolicibacterium sphagni* DSM 44076 | AF547877.1 | FR733719.1 | NOZR01000019.1 |
| *Mycobacterium stomatepiae* DSM 45059 | AM902968.1 | AM884331.1 | HM022213.1 |
| *Mycolicibacter terrae* FI-07146 | JN571207.1 | JN571181.1 | JN571265.1 |
| *Mycolicibacterium thermoresistibile* DS38 | AF547880.1 | KU362966.1 | KU363003.1 |
| *Mycobacterium triplex* ATCC 700071 | GQ153291.1 | NR117226.1 | GQ153311.1 |
| *Mycobacterium tuberculosis* H37rv | AL123456.3 | AL123456.3 | AL123456.3 |
| *Mycolicibacterium vaccae* ATCC 15483 | AY299163.1 | NR041899.1 | JF923624.1 |
| *Mycolicibacter virginiensis* MO-233 | JX154109.1 | KR025879.1 | KR025885.1 |
| *Mycobacterium vulneris* NLA000700772 | EU834054.1 | EU834055.1 | EU834057.1 |
| *Mycobacterium xenopi* ATCC 19250 | AY373454.1 | MH169241.1 | AF060365.1 |
| *Mycobacterium yongonense* 05 1319 | JN605801.1 | JF738056.1 | JF738053.1 |
