# Supplementary figures and images for: Diversity of Mycobacteriaceae from aquatic environment at the São Paulo Zoological Park Foundation in Brazil
Source: PLoS One. 2020 Jan 14;15(1):e0227759. doi: 10.1371/journal.pone.0227759 (PMC6959594; doi:10.1371/journal.pone.0227759)

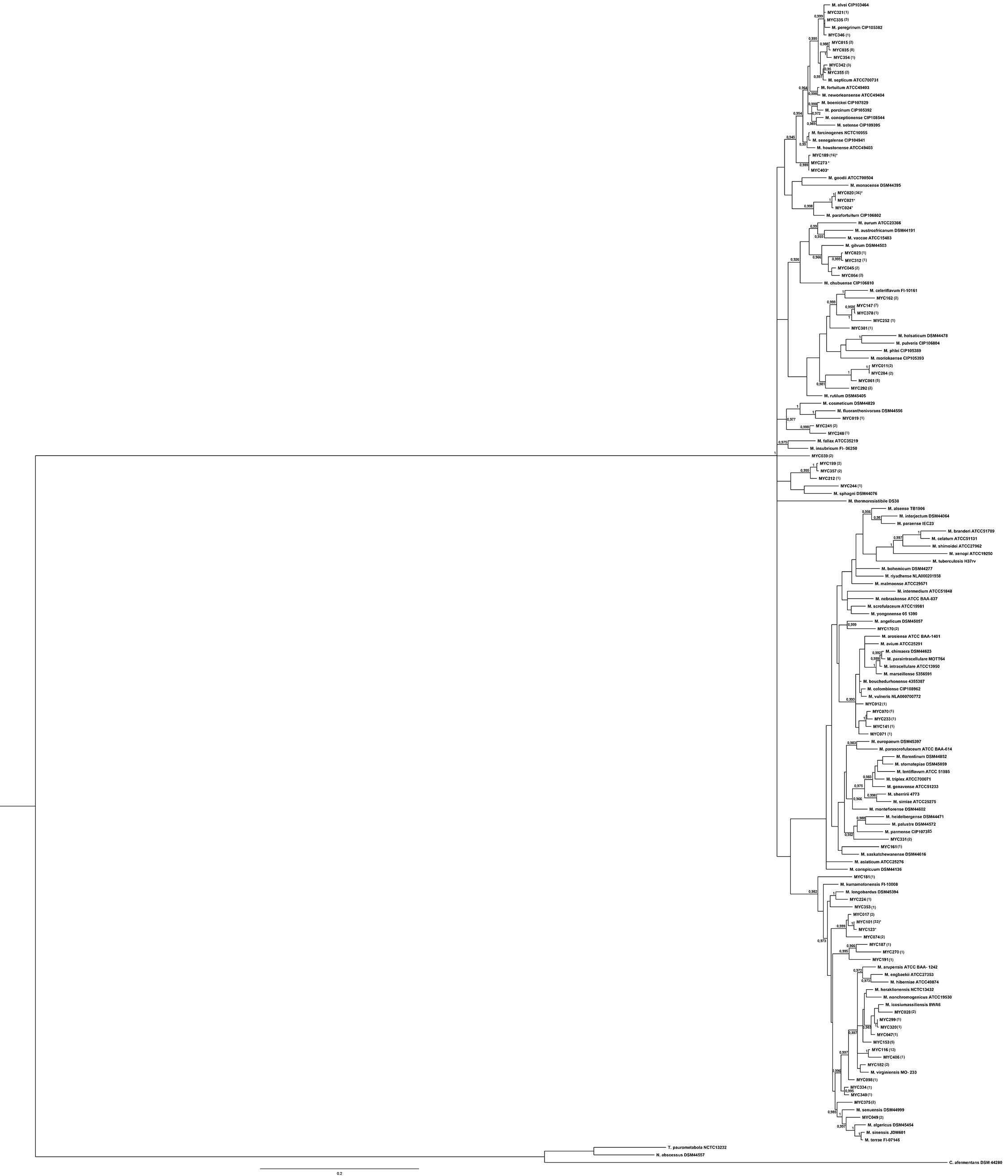

Supplement: S1 Fig — Numbers above branches are Bayesian posterior probabilities (≥0.95). (TIF) [file pone.0227759.s002.tif]
